# Characterization of the effects of immunomodulatory drug fingolimod (FTY720) on human T cell receptor signaling pathways

**DOI:** 10.1038/s41598-018-29355-0

**Published:** 2018-07-19

**Authors:** Alan Baer, Winston Colon-Moran, Nirjal Bhattarai

**Affiliations:** Division of Cellular and Gene Therapies, Center for Biologics Evaluation and Research, Food and Drug Administration, Silver Spring, Maryland 20993 USA

## Abstract

Immune responses against gene therapy products limit its therapeutic efficacy and present a safety risk. Identification of agents that blunt immune reactions may aid in developing novel immunomodulatory therapies. Fingolimod (FTY720) is an FDA approved immunomodulatory drug for treating multiple sclerosis that inhibits lymphocyte egress from lymphoid tissues by down regulating sphingosine-1 phosphate receptor (S1PR). Recent studies found that FTY720 inhibits T cell activation (TCA) in a S1PR-independent manner; however, the mechanism is incompletely understood. Here we characterized the effects of FTY720 on human T cell receptor (TCR) signaling pathways. FTY720 inhibited both the TCR-dependent and independent activation of primary human T cells. FTY720 did not affect proximal TCR signaling events as measured by phosphorylation of Lck, ZAP-70 and LAT; however, inhibited PMA/Ionomycin induced distal TCR signaling as measured by IL-2, IFN-γ release and CD25 expression. FTY720 induced aberrant NFAT1, AP1 and NFκB activation which were associated with increased acetylation of histone (H3K9). Phosphorylated FTY720 did not inhibit TCA, and arachidonic acid did not rescue FTY720 mediated inhibition of TCA. These data suggest that FTY720 mediated inhibition of TCA is due to inhibition of distal TCR signaling. Understanding FTY720-mediated inhibition of TCA may aid in developing novel FTY720-based immunomodulatory agents.

## Introduction

Gene therapies utilizing viral vectors have the potential to treat many human diseases^1,2^. Although these therapies hold great promise, host immune responses to viral vectors and their components greatly limit the efficacy of these therapies and present a significant safety risk^3–5^. Immunosuppressive agents like corticosteroids or inhibitors that target IL-6 signaling pathways are commonly used to reduce host immune responses and inflammation; however, use of these agents can be problematic since they are non-specific, have heterogeneous clinical responses, and there are significant numbers of non-responders^6,7^. Thus, development of novel immunomodulatory agents that selectively blunt T cell responses or T cell associated inflammation may greatly benefit subjects receiving these therapies.

Fingolimod (FTY720) is an FDA approved immunosuppressive drug used for the treatment of a relapsing and remitting form of multiple sclerosis (MS)^8–10^. The primary mechanism of immunosuppression is FTY720 induced lymphopenia. Upon cellular adsorption, FTY720 is phosphorylated by sphingosine kinases into its active state^9^. Phosphorylated FTY720 (pFTY720) then downregulates the sphingosine 1 phosphate receptor (S1PR) and inhibits lymphocyte egress from the thymus and secondary lymphoid organs, resulting in a reduction of peripheral lymphocytes^8^. Recent studies have found that FTY720 directly inhibits T cell activation in a S1PR independent manner^10,11^; however, the mechanism for this is incompletely understood. In T cells, FTY720 inhibits cytosolic phospholipase A2α (cPLA2α), which regulates arachidonic acid (AA) release and its subsequent synthesis into eicosanoids^10,12^. Exogenous addition of AA was found to partially rescue FTY720 mediated inhibition of CD8 T cell function in murine splenocytes, suggesting that FTY720 inhibits T cell function in part due to the inhibition of AA synthesis^10^. While this pathway is activated in response to cytokines and intracellular calcium and regulates T cell function, the effect of AA on FTY720 mediated inhibition of human T cell function is unknown. Furthermore, FTY720 induces expression of T cell factor 1 (TCF-1), which inhibits expression of some but not all inflammatory genes by binding to their promoter/enhancer regions^11^. These studies suggest that FTY720 inhibits human T cell function by various mechanisms. T cell receptor (TCR) signaling is required for T cell activation and function; however, the effect of FTY720 on human TCR signaling pathways has not been studied. Here we characterized the effects of FTY720 on human TCR signaling to gain novel insights into the mechanism of FTY720 mediated inhibition of T cell function.

FTY720 inhibited both TCR-dependent and TCR-independent T cell activation in primary human T cells in a dose-dependent manner. While FTY720 did not affect activation of proximal TCR-induced signaling events, it inhibited distal TCR signaling induced by PMA/Ionomycin. The inhibition of distal TCR signaling was not due to the effects of FTY720 on distal transcription factors NFAT1, NFκB and AP1 expression; however, it induced specific epigenetic modifications of the histone H3 protein in human T cells which was associated with aberrant activation of NFAT1, NFκB and AP1-dependent reporter genes. Furthermore, the phosphorylated form of FTY720 did not affect distal TCR signaling, and administration of AA did not rescue FTY720-mediated inhibition of human T cell activation. Together, these data provide novel insights into the effects of FT720 on human TCR signaling pathways, and suggest that FTY720 inhibits distal TCR signaling in a cPLA2α and S1PR independent manner.

## Results

### FTY720 inhibits T cell receptor (TCR) mediated T cell activation in primary human T cells

**Figure 1:**
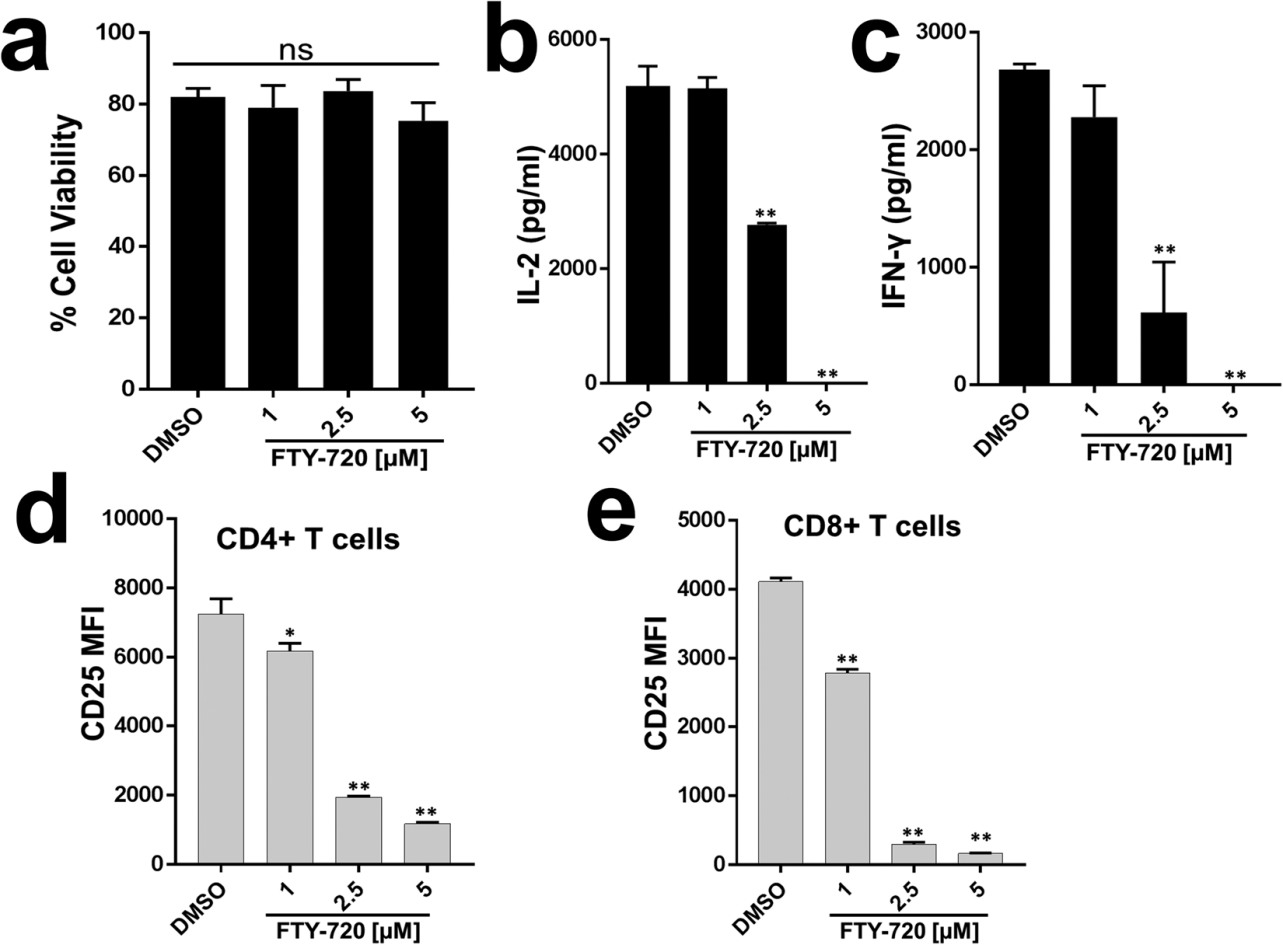
FTY720 inhibits T cell receptor (TCR) mediated T cell activation in primary human T cells. (**a**) Cell viability was measured in primary human T cells following 24 hours of treatment with various concentrations of FTY720 or DMSO by a trypan blue exclusion method. Primary human T cells were treated with DMSO or FTY720, and following TCR engagement with anti-CD3/CD28, T cell activation was measured by assessing **(b)** IL-2, (**c**) IFN-γ release and CD25 surface expression on (**d**) CD4 and (**e**) CD8 T cells. MFI = mean fluorescence intensity, ns = not significant. Data represent the average of three technical replicates, and the standard deviation is shown. Each experiment was independently performed with three different donors with similar results. *P < 0.05; **P < 0.01.

The primary mechanism of FTY720 mediated immunomodulation is downregulation of the S1PR receptor by phosphorylated FTY720, which inhibits lymphocyte egress from the thymus and secondary lymphoid organs resulting in lymphopenia^8^. Recent studies found that FTY720 directly inhibits T cell function independently of the S1PR pathway^10–12^; however, the mechanism is not completely understood. Since T cell receptor (TCR) signaling and T cell activation (TCA) is required for T cell function and the effect of FTY720 on human TCR signaling pathways has not been studied, we assessed the effect of FTY720 on TCR-mediated TCA. Primary human T cells obtained from healthy donors were treated with FTY720 or DMSO (vehicle) as a control. FTY720 did not affect cellular viability as measured by a trypan-blue exclusion assay (Fig. 1a). Following TCR engagement with anti-CD3/CD28, TCA was assessed. FTY720 inhibited TCR-induced IL-2 (Fig. 1b) and IFN-γ (Fig. 1c) release in a dose dependent manner. Upon TCA, surface expression of CD25, alpha chain of the IL-2 receptor, is induced. FTY-720 inhibited CD25 expression in both CD4 and CD8 T cells (Fig. 1d,e). Phosphorylated FTY720 (pFTY720) did not affect IL-2, IFN-γ and CD25 expression in primary human T cells (Suppl. Fig. S1).

Together, these data suggest that FTY720 inhibits TCR-induced TCA in primary human T cells.

### FTY720 does not affect proximal TCR signaling events

**Figure 2:**
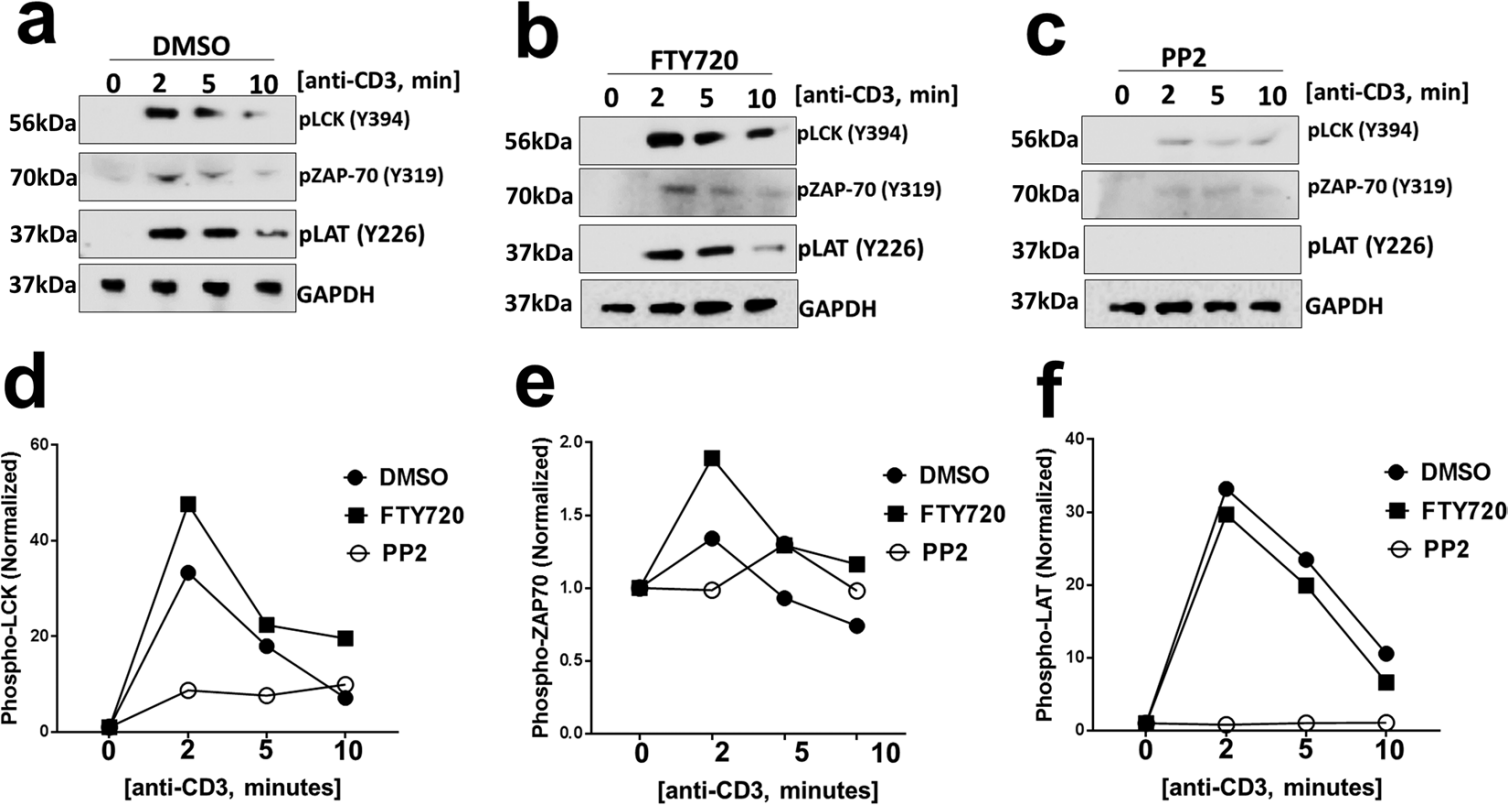
FTY720 does not affect proximal T cell receptor (TCR) signaling events. Activation of proximal TCR signaling events was measured in unstimulated (0) or anti-CD3 stimulated (2, 5 and 10 minutes) primary human T cells by assessing phosphorylation of Lck at tyrosine 394 (Y394), ZAP-70 at Y319 and LAT at Y226, following (**a**) DMSO or (**b**) FTY720 (5 µM) or (**c**) Src-kinase inhibitor (PP2, 5 µM) treatment for 1 hour at 37 °C. GAPDH protein expression was measured as a loading control. Quantification of immunoblots for **(d)** phospho-Lck, **(e)** phospho-ZAP70, and (**f**) phospho-LAT are shown. Phospho proteins levels were normalized to GAPDH. Each experiment was independently performed using primary human T cells from two different donors with similar results.

Engagement of the TCR with a peptide-bound MHC complex present on the surface of the antigen-presenting cells activates proximal TCR signaling events^13,14^. One of the earliest events is the activation of lymphocyte-specific tyrosine kinase (Lck) by trans-auto-phosphorylation at tyrosine 394 (Y394)^15^. To assess the effect of FTY720 on Lck Y394 phosphorylation and activation, primary human T cells were treated with DMSO control or FTY720 or Src-kinase inhibitor (PP2) for 1 hour. Following TCR engagement with anti-CD3, phosphorylation of Lck Y394 was measured at three different time points by immunoblots. Lck Y394 phosphorylation did not change in T cells treated with FTY720 compared to the DMSO control (Fig. 2a,b,d). PP2 treatment was used as a positive control for inhibition of proximal TCR signaling. As expected, PP2 treated cells had reduced levels of Lck Y394 (Fig. 2c,d).

Upon activation, Lck phosphorylates the immunoreceptor tyrosine-based activation motif (ITAM) on the zeta-chain of the TCR^15,16^. Phosphorylated ITAM serves as a docking site for the ZAP-70 tyrosine kinase. Upon binding to the ITAM motif, ZAP-70 is activated by Lck-mediated phosphorylation. Phosphorylation of ZAP-70 is an important step in proximal TCR signaling. ZAP-70 phosphorylation was not affected in T cells treated with FTY720 compared to the DMSO control (Fig. 2a,b,e).

Next, we assessed phosphorylation of the transmembrane adaptor protein LAT, linker for activation of T cells. Activated ZAP-70 tyrosine kinase phosphorylates LAT, and LAT phosphorylation is required to form a multiprotein complex that amplifies proximal TCR signals^14–16^. Consistent with the results obtained with Lck and ZAP-70, FTY720 treatment did not affect LAT phosphorylation in human T cells following TCR engagement (Fig. 2a,b,f). In all cases, PP2 treatment reduced proximal TCR signaling (2c, 2d, 2e, 2 f).

Together, these data suggest that FTY720 does not affect activation of the proximal TCR signaling events.

### FTY720 inhibits distal TCR signaling events in primary human T cells

**Figure 3:**
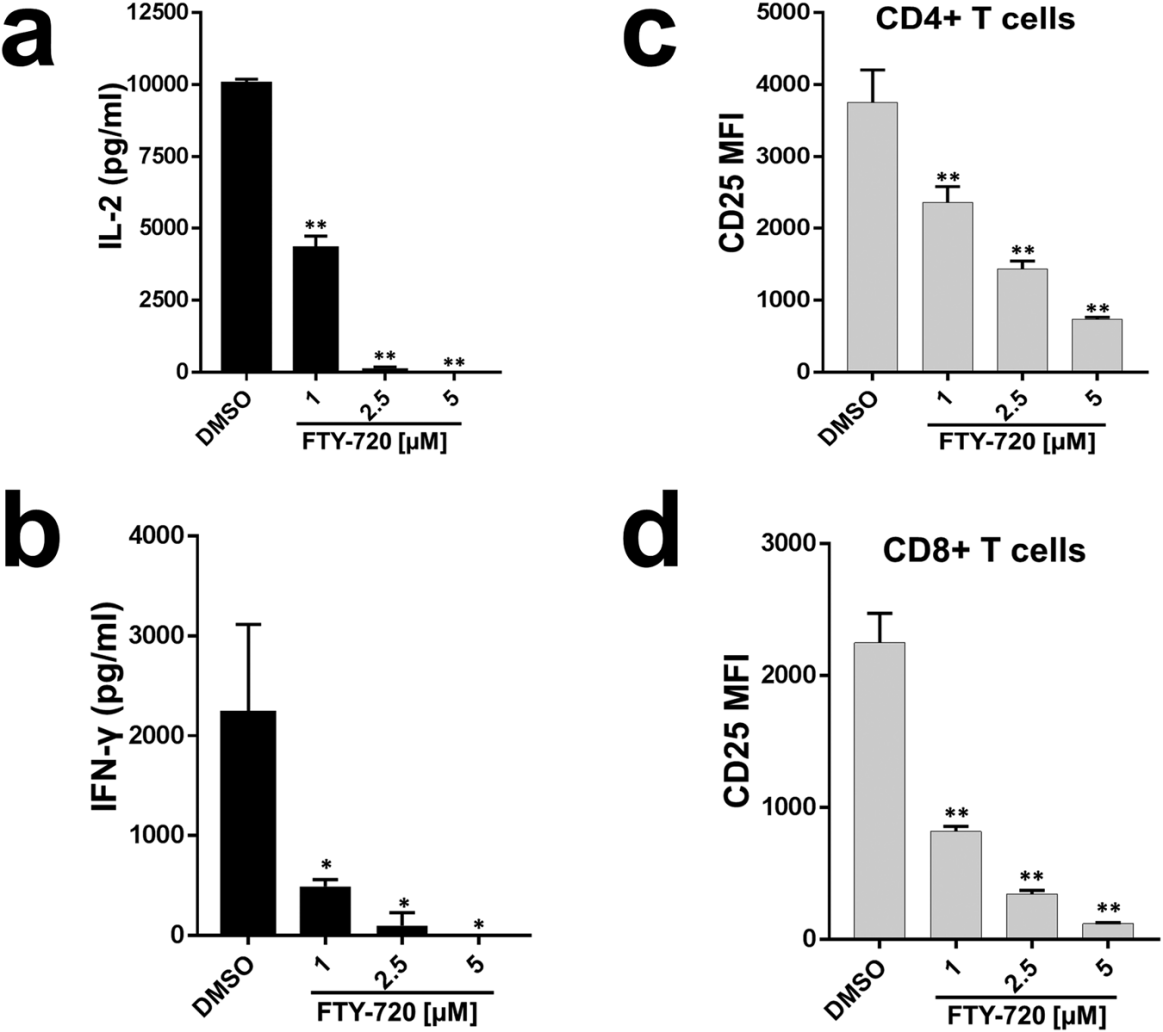
FTY720 inhibits distal TCR signaling events in primary human T cells. Primary human T cells were treated with DMSO or FTY720. Following stimulation with PMA/Ionomycin, distal TCR signaling mediated T cell activation was measured by assessing (**a**) IL-2, (**b**) IFN-γ release and CD25 surface expression on (**c**) CD4 and (**d**) CD8 T cells. MFI = mean fluorescent intensity. Data represents the average of three technical replicates, and the standard deviation is shown. Each experiment was independently performed using three different donors with similar results. *P < 0.05; **P < 0.01.

T cell activation (TCA) with phorbol 12-myristate 13-acetate (PMA) and ionomycin bypasses activation of proximal TCR signaling events, and activates distal TCR signaling pathways through activation of protein kinase C (PKC) and calcineurin^17–19^. To assess the role of FTY720 on distal TCR signaling, primary human T cells were treated with various concentrations of FTY720 or phosphorylated FTY720 (pFTY720). TCA was measured before and following PMA/Ionomycin (P/I) stimulation. In absence of P/I, IL-2, IFN-γ, and CD25 expression were not affected by FTY720 and pFTY720 (Suppl. Fig. S2). FTY720 inhibited P/I induced TCA in a dose-dependent manner compared to the DMSO control as measured by IL-2 (Fig. 3a) and IFN-γ (Fig. 3b) release, and CD25 expression on CD4 and CD8 T cells (Fig. 3c,d). pFTY720 did not affect P/I induced TCA (Suppl. Fig. S3). Furthermore, the inhibitory effect of FTY720 was still present in T cells treated with calcineurin inhibitors, cyclosporin A (CsA) and FK560 (Suppl. Fig. S3).

These data suggest that FTY720 inhibits TCA by interfering with distal TCR signaling event(s) either at or downstream of calcineurin and PKC activation.

### FTY720 induces aberrant NFAT1, NFκB and AP1 activation

**Figure 4:**
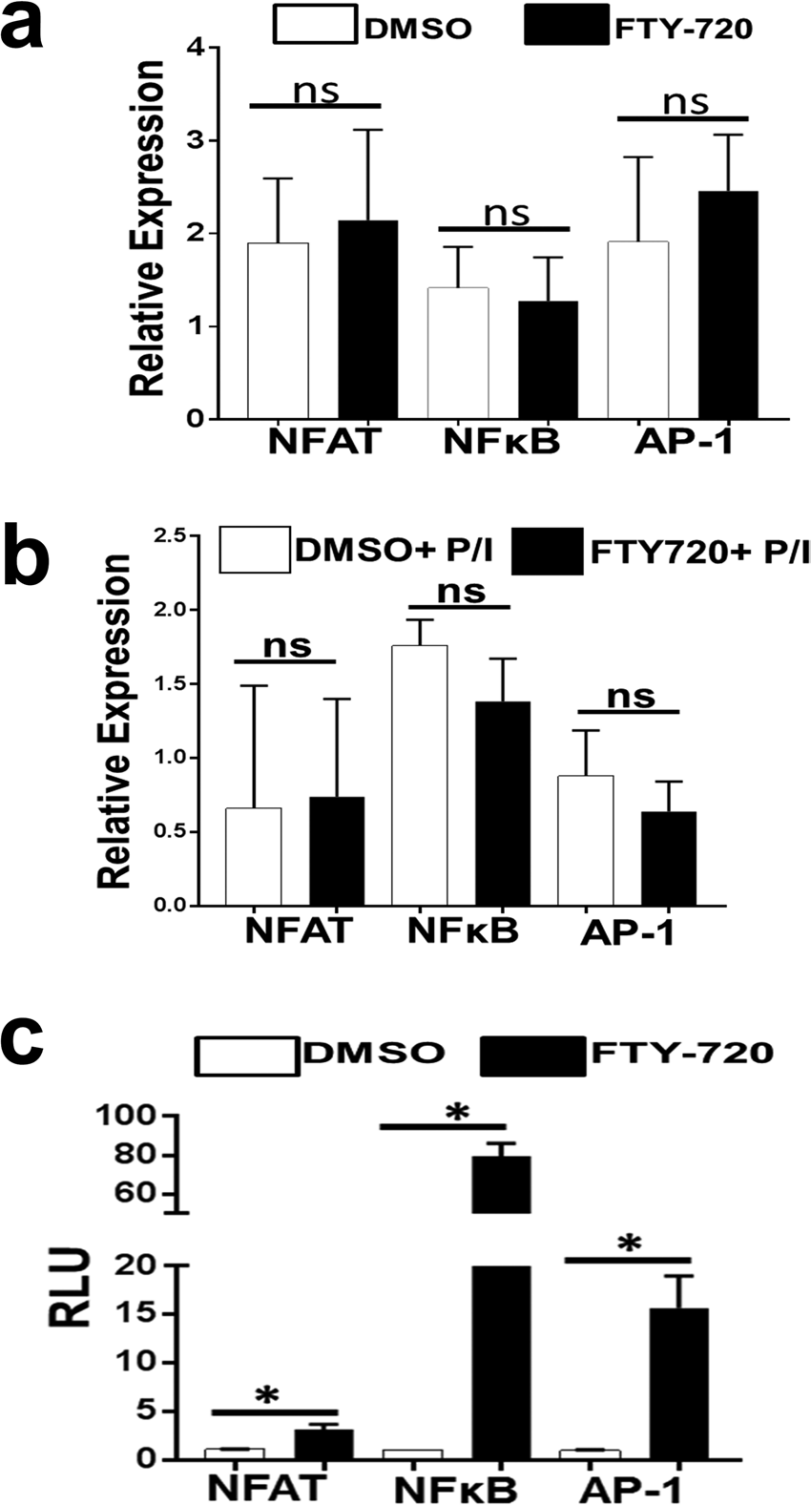
FTY720 induces aberrant NFAT1, NFκB and AP1 activation. Immunoblot analysis of NFAT1, NFκB and AP1 protein expression in **(a)** unstimulated or (**b**) PMA/Ionomycin (P/I) stimulated primary human T cells following 24 hours treatment with DMSO or FTY720 (5 µM). Actin protein expression was measured as a loading control and was used to calculate relative expression. (**c**) NFAT1, NFκB and AP1 dependent firefly luciferase activity was measured in Jurkat T cells following 24 hours treatment with DMSO or FTY720 (5 µM). Firefly luciferase activity was normalized to renilla luciferase activity. Data represents the average of three technical replicates, and the standard deviation is shown. Protein expression was measured in human T cells obtained from three donors and average is shown. Luciferase experiment was independently performed three times with similar results. RLU = relative luciferase units, ns = not significant. *P < 0.01.

In resting human T cells, the transcription factors NFAT1, NFκB and AP1 are present in an inactive form and reside in the cytoplasmic compartment^19^. Following activation of distal TCR signaling, these transcription factors translocate to the nucleus and transcribe TCR-induced genes^20,21^. Since FTY720 has differential effects on cellular gene expression^11^ and inhibits distal TCR signaling, we assessed the effect of FTY720 on NFAT1, NFκB and AP1 expression. Unstimulated or PMA/Ionomycin (P/I) stimulated primary human T cells were treated with FTY720 or DMSO, and protein expression was measured by immunoblots. FTY720 did not affect NFAT1, NFκB or AP1 protein expression in unstimulated (Fig. 4a) or P/I stimulated human T cells (Fig. 4b). This suggests that the mechanism of FTY720 mediated inhibition of distal TCR signaling is not due to its effect on expression of these essential transcription factors.

As noted, upon activation of distal TCR signaling these transcription factors activate target gene expression. To assess the effect of FTY720 on the transcriptional activity of these transcription factors, Jurkat T cells were transfected with either NFAT1, AP1 or NFκB dependent luciferase reporter plasmids, along with a renilla luciferase control plasmid. Following 24 hours of treatment with either DMSO control or FTY720, luciferase activity was measured. Surprisingly, NFAT1, AP1 and NFκB dependent luciferase activity was significantly higher in FTY720 treated cells compared to the control (Fig. 4c). This suggests that FTY720 induces aberrant activation of NFAT1, AP1 and NFκB in human T cells without affecting their steady state protein expression.

Next, we assessed aberrant activation and nuclear translocation of NFAT1, AP1 and NFκB in primary human T cells. DMSO or FTY720 treated primary human T cells were either stimulated with PMA/Ionomycin (P/I) or left unstimulated. Nuclear proteins were extracted as described previously^17^ and assessed for NFAT1, AP1 and NFκB. FTY720 treatment increased nuclear translocation of these transcription factors compared to the DMSO control (Suppl. Fig. S4). As expected, T cells stimulated with P/I had higher levels of nuclear NFAT, AP1 and NFκB which was further enhanced by FTY720 (Suppl. Fig. S4). These data are consistent with the observation that FTY720 induces aberrant activation of NFAT1, AP1 and NFκB in Jurkat T cells (Fig. 4c), and suggest that FTY720 induces aberrant activation of these transcription factors in primary human T cells.

### FTY720 induces epigenetic modification of histone protein in primary human T cells

**Figure 5:**
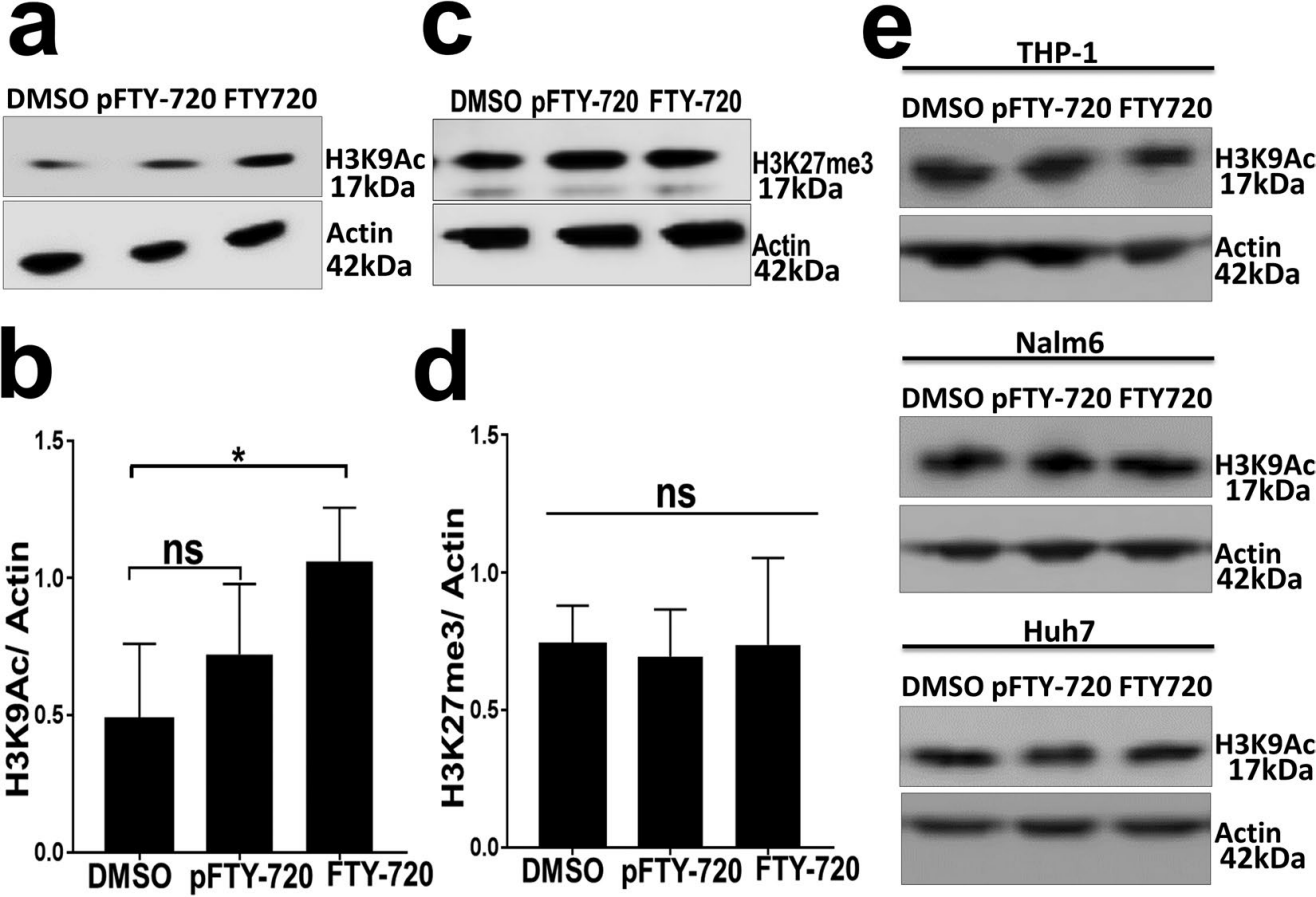
FTY720 induces epigenetic modification of histone protein in primary human T cells. Immunoblot analysis of (**a**) histone H3 lysine 9 acetylation (H3K9Ac) and (**c**) histone H3 lysine 27 tri-methylation (H3K27me3) in primary human T cells following 24 hours of treatment with DMSO, phospho FTY720 (pFTY720, 5 µM) or FTY720 (5 µM). Actin protein expression was used as a loading control. Quantification of histone blots (**b**) H3K9Ac and (**d**) H3K27me3 were performed in primary human T cells from five healthy donors after treatment as described in panels (**a**,**c**). (**e**) Immunoblot analysis of H3K9Ac in three different human cell lines, THP1, Nalm6 and Huh7 after treatment as described in panel (**a**). Amount of histone H3 protein was normalized to the respective actin protein. ns = not significant, *P < 0.05.

A previous study found that FTY720 induces chromatin modifications such as tri-methylation of lysine 9 and lysine 27 of histone H3 on the interferon-gamma promoter which contributes to its immunosuppressive effect^11^. To better understand the mechanism for aberrant transcriptional activity of NFAT1, AP1 and NFκB in FTY720 treated human T cells, we assessed two specific epigenetic markers on histone H3 following FTY720 treatment in primary human T cells. Acetylation of lysine 9 (H3K9Ac) on histone H3 is associated with gene expression, and tri-methylation of lysine 27 (H3K27me3) is associated with gene repression^22,23^. In primary human T cells treated with FTY720, the level of H3K9Ac was significantly higher compared to the control DMSO treated cells (Fig. 5a,b); however, it did not affect the level of H3K27me3 compared to the DMSO control (Fig. 5c,d).

Phosphorylation of FTY720 is required for downregulation of sphingosine 1 phosphate receptor (S1PR)^9^ and previous studies have found that FTY720 directly inhibits T cell activation in a S1PR independent manner^10,11^. To assess if phosphorylation of FTY720 (pFTY720) was required for observed epigenetic modifications of histone H3 in human T cells, the levels of H3K9Ac and H3K27me3 were assessed in primary T cells treated with phosphorylated FTY720 (pFTY720) and compared with DMSO treated cells. pFTY720 did not significantly affect H3K9Ac and H3K27me3 (Fig. 5b,d). To assess if the inability of pFTY720 to induce H3K9Ac was due to lack of cellular penetration of pFTY720 compared to FTY720, primary human T cells were treated with sphingosine kinase inhibitor (SphK) in the presence of pFTY720 and FTY720. We found that primary human T cells treated with SphK had increased surface expression of sphingosine-1-phosphate receptor 1 (S1PR1) compared to the untreated control cells (Suppl. Fig. S5). Both pFTY720 and FTY720 inhibited Sphk inhibitor mediated S1PR1 upregulation suggesting that both FTY720 and pFTY720 can inhibit intracellular pathways that upregulate S1PR1 expression in presence of SphK inhibitor.

Next, we assessed if the increase in H3K9Ac induced by FTY720 was specific to human T cells. Three different human cell lines, THP-1 (monocyte-derived), Nalm6 (pre-B cell-derived) and Huh7 (hepatocyte-derived), were treated with DMSO, pFTY720 or FTY720. FTY720 did not affect H3K9Ac in these three different human cell lines (Fig. 5e).

Together, these data suggest that FTY720 specifically induces histone H3 acetylation (H3K9Ac) in human T cells, a marker associated with gene activation which may contribute to the aberrant NFAT1, AP1 and NFκB dependent gene expression independently of its effect on the S1PR pathway.

### Arachidonic acid does not rescue FTY720 mediated inhibition of human T cell activation

**Figure 6:**
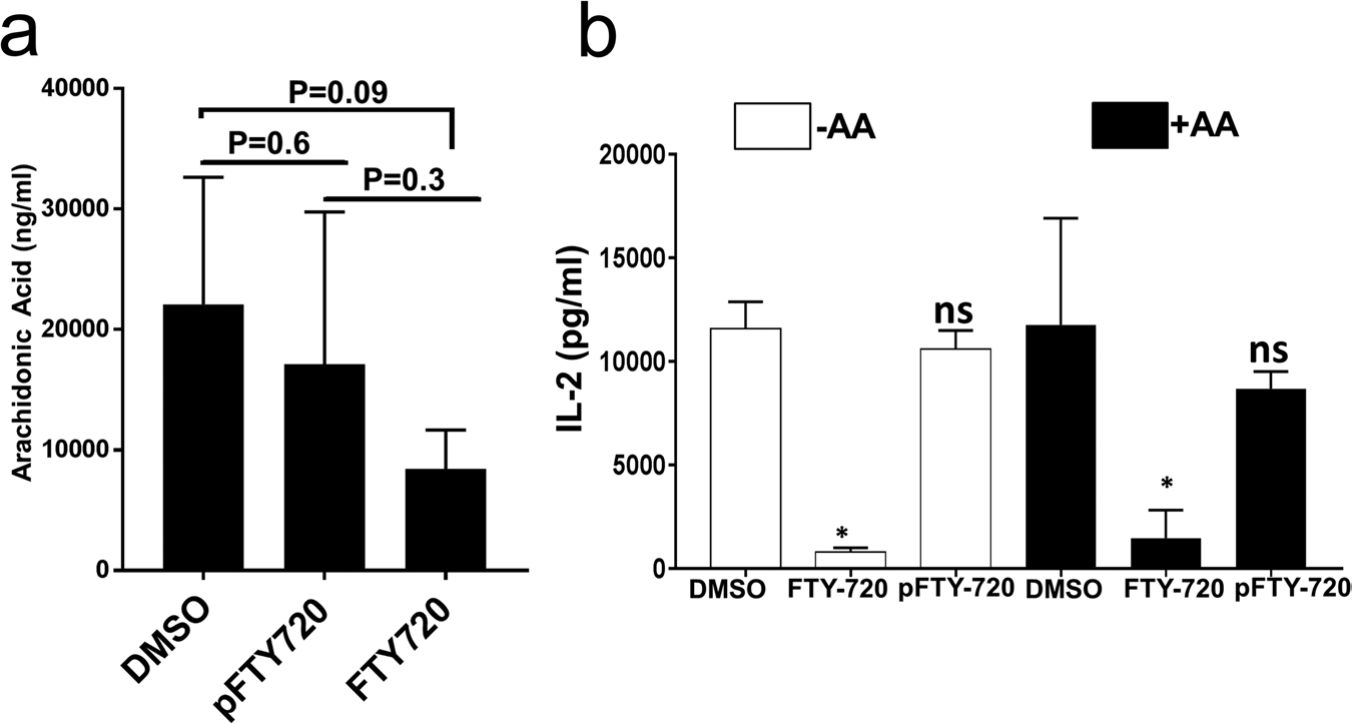
Arachidonic acid does not rescue FTY720 mediated inhibition of human T cell activation. (**a**) Primary human T cells were treated with DMSO, phospho FTY720 (pFTY720, 5 µM) or FTY720 (5 µM). Following 24 hours stimulation with PMA/Ionomycin, arachidonic acid was measured in culture supernatant. **(b)** Primary human T cells were treated with either DMSO, phospho FTY720 (pFTY720, 5 µM) or FTY720 (5 µM) in the presence (+AA, 30 µM) or absence of arachidonic acid (-AA) for 24 hours. Following stimulation with PMA/Ionomycin, distal TCR signaling mediated T cell activation was assessed by measuring IL-2 release. Data represents the average of three technical replicates, and the standard deviation is shown. Experiment was performed in T cells obtained from two donors with similar results. ns = not significant, *P < 0.01.

Previous studies found that FTY720 inhibits cytosolic phospholipase A2 (cPLA2α) which catalyzes the hydrolysis of the membrane glycerophospholipids to release arachidonic acid (AA), a precursor for prostaglandins and leukotrienes^10,12,24^. Prostaglandins play an important immunoregulatory role during TCA^25^. In murine CD8 T cells, exogenous addition of AA was shown to partially rescue FTY720 mediated inhibition of CD8 T cell function^10^, suggesting that the inhibitory effect of FTY720 on murine CD8 T cells is mediated in part by its effect on AA synthesis. The effect of AA on FTY720 mediated inhibition of human TCA is unknown. First, we assessed the effect of FTY720 on AA synthesis. Primary human T cells were treated with DMSO, phosphorylated FTY720 (pFTY720) or FTY720. Following PMA and Ionomycin (P/) stimulation, AA was measured in the culture supernatant. The amount of AA released in the culture supernatant was lower but not significantly different in FTY720 treated cells compared to the DMSO treated cells (Fig. 6a). pFTY720 did not affect AA release compared to the DMSO control (Fig. 6a).

To determine if exogenous AA rescues the observed FTY720 mediated inhibition of human TCA, primary human T cells were treated with DMSO, pFTY720 or FTY720 in the presence or absence of AA. Following activation of distal TCR signaling with P/I, TCA was measured by assessing IL-2 release. As expected FTY720 significantly inhibited TCA in the absence of AA; however, exogenous AA did not rescue FTY720 mediated inhibition of human TCA (Fig. 6b). Furthermore, pFTY720 did not inhibit TCA in the presence or absence of AA (Fig. 6b).

Together, these data suggest that the mechanism of FTY720 mediated inhibition of human TCA is independent of its effect on AA synthesis. Consistent with the previous observation (Fig. 5), pFTY720 did not affect human TCA further confirming that FTY720 inhibits TCA in a S1PR independent manner.

## Discussion

T cell receptor (TCR) activation and subsequent TCR signaling is required for generation of an effective T cell response^26,27^. In gene therapy, chronic TCR signaling and T cell responses against therapeutic products may lead to increased T cell associated inflammation and reduced efficacy^4,28^. In this study, we characterized the effects of the immunomodulatory drug fingolimod (FTY720) on human TCR signaling pathways and TCA to gain insights into its mechanism of immunosuppression.

We found that FTY720 inhibited TCR-dependent TCA in primary human T cells (Fig. 1). FTY720 did not affect activation of proximal TCR signaling events (Fig. 2), suggesting inhibition was likely due to the effect of FTY720 on distal TCR signaling pathway. Consistent with this, we found that FTY720 inhibited TCR-independent distal TCR signaling and TCA in primary human T cells (Fig. 3). Although FTY720 has been shown to cause differential gene expression, the effect of FTY720 on distal TCR signaling was not due to changes in the expression of NFAT1, AP1 or NFκB (Fig. 4a,b). However, FTY720 treatment induced aberrant activation of NFAT1, AP1 and NFκB resulting in increased transcriptional activity (Fig. 4c), which was associated with an increased level of histone H3 lysine 9 acetylation (H3K9Ac), a marker for active gene expression (Fig. 5a,b). Conversely, we did not find any differences in the level of histone H3 lysine 27 tri-methylation (H3K27me3), a marker for gene repression in FTY720 treated cells compared to the DMSO treated cells (Fig. 5c,d). Furthermore, the level of histone H3K9Ac was not significantly higher in primary T cells treated with phosphorylated FTY720 (pFTY720) when compared to the DMSO control (Fig. 5a,b). The molecular mechanisms for activation of NFAT1, AP1 and NFκB are complex^21,29,30^. FTY720 may also affect cellular factors responsible for activating these transcription factors. Future studies into the effects of FTY720 on regulatory factors activating NFAT1, AP1 and NFκB may provide additional insights into the mechanism for FTY720 mediated aberrant activation of these transcription factors.

A previous study found that FTY720 has differential effects on gene expression^11^. FTY720 induced specific epigenetic changes in the interferon gamma and granzyme B promoter region. Although we did not assess the effect of FTY720 on epigenetic markers in these promoter regions, the fact that an epigenetic marker of active gene expression (H3K9Ac) was induced in FTY720 treated cells is consistent with this prior study. Furthermore, increased H3K9Ac is associated with gene expression^22,23^. The increased level of FTY720-induced H3K9Ac likely contributes to aberrant activation of NFAT1, AP1 and NFκB dependent target gene expression resulting into inhibition of TCA. Current studies are underway to characterize the role of increased H3K9Ac in aberrant activation of these transcription factors and T cell inhibition. Although we did not find changes in the level of H3K27me3 in FTY720 treated cells, we did not study other epigenetic markers of gene repression. Since pFTY720 had no significant effect on H3K9Ac, and did not inhibit distal TCR signaling mediated TCA (Figs 3, 5 and 6), these data further support the findings that FTY720 inhibits TCA in a S1PR independent manner^10,11^.

Another study also found that in murine CD8 T cells exogenous addition of arachidonic acid (AA) partially rescues FTY720 mediated inhibition of CD8 T cell function^10^. We found that the inhibitory effect of FTY720 on distal TCR signaling mediated human TCA was not rescued by addition of AA (Fig. 6) suggesting a novel cPLA2α independent mechanism of inhibition. It is not known if FTY720 causes similar epigenetic changes in murine T cells as observed in the human T cells, which may explain the inability of AA to rescue human TCA during FTY720 treatment. Future studies comparing specific epigenetic changes in human and murine T cells following FTY720 treatment may provide additional insights on these discrepancies.

**Figure 7:**
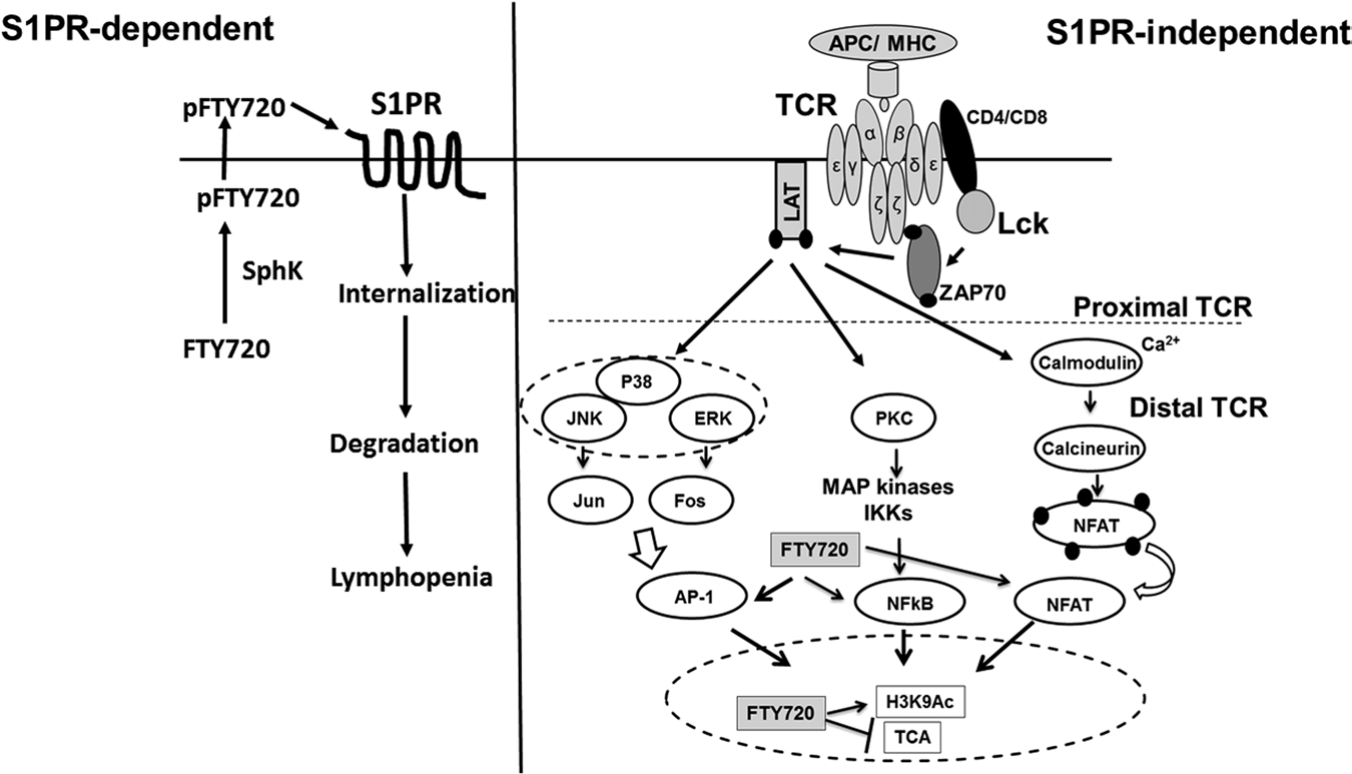
Proposed model for mechanisms of FTY720 mediated inhibition of T cell function. S1PR-dependent mechanism: Upon cellular entry FTY720 is phosphorylated by cellular sphingosine kinases (SphK). Phosphorylated FTY720 (pFTY720) binds to sphingosine-1-phosphate receptor (S1PR) which results into receptor internalization, and degradation. Lack of S1PR receptor expression inhibits lymphocytes to egress out of lymphoid tissues resulting into lymphopenia and loss of peripheral T cell function. S1PR-independent mechanism: Engagement of the T cell receptor (TCR) with a peptide bound MHC complex present on the surface of antigen-presenting cells (APC) initiates proximal TCR signaling events, resulting in activation of Lck, ZAP-70 and LAT protein. Activation of proximal TCR signaling factors culminates in activation of distal TCR signaling events such as activation and nuclear translocation of the transcription factors NFAT1, NFκB and AP1 that results in T cell activation. FTY720 induces aberrant nuclear translocation and activation of NFAT1, NFκB and AP1, and increases acetylation of histone H3 lysine 9 (H3K9Ac), a marker of active gene transcription. The aberrant activation of NFAT1, NFκB and AP1 by FTY720 results in inhibition of T cell activation (TCA).

In conclusion, our study characterized the effects of FTY720 on human TCR signaling pathways, and suggests that in addition to the S1PR-dependent modulation of T cell response, FTY720 also causes aberrant activation of NFAT1, AP1 and NFκB and induces epigenetic changes in human T cells resulting into inhibition of T cell activation (Fig. 7). These data provide novel insights into the immunomodulatory effects of FTY720 on TCA. Understanding the mechanism of FTY720 mediated inhibition of TCR signaling may aid in developing novel FTY720 based immunomodulatory agents that selectively blunt T cell responses against gene therapy products by selectively targeting TCR signaling.

## Methods

### Peripheral blood mononuclear cells (PBMCs)

Whole blood was obtained from healthy donors at the National Institutes of Health (NIH) Blood Bank. All the donors provided written consent for their blood products to be used in research projects. All the samples provided to the investigators were de-identified. This study was exempted by the FDA’s IRB, Research Involving Human Subject Committee (RIHSC). PBMCs were isolated from whole blood using Ficoll-Hypaque gradient centrifugation. Isolated PBMCs were washed in cold PBS, and resuspended in complete RPMI and incubated overnight at 37 °C with 5% CO_2_.

### Cells

Jurkat T cell line (clone E6.1), and THP-1 cells was obtained from ATCC and cultured as suggested by ATCC. Nalm6 and Huh7 cells were gifts from Drs. Steven Bauer (FDA) and Dino Figelstock (FDA) respectively. Cells were maintained in either RPMI 1640 or DMEM supplemented with 10% heat-inactivated fetal calf serum (hFBS), 2 mM L-glutamine, 100 IU/ml penicillin, and 100 µg/ml streptomycin. Cell viability was determined using a trypan blue exclusion method and counted using the Countess II FL automated cell counter (Invitrogen).

### Drug Compounds

Fingolimod (FTY720) and FTY720 Phosphate (pFTY720) were purchased from Cayman Chemical, Arachidonic Acid from MP Biomedicals, dimethyl sulfoxide (DMSO) from Thermo Fisher, sphingosine kinase inhibitor (SKI II) from Tocris, Src-kinase inhibitor PP2 and calcineurin inhibitors FK506 and Cyclosporin A from Selleck Chemicals.

### Cell Stimulation

T cells (1 × 10^6^ cells/ml) were treated with the inhibitors as indicated. T cell receptor (TCR) dependent activation was measured following stimulation with anti-CD3/CD28 (100 ng/ml). TCR-independent activation was measured following stimulation with phorbol-12-myristate 13-acetate (PMA, 50 ng/ml) and ionomycin (1 µg/ml). Following 24 hours of stimulation, cellular receptor expression and cytokines were measured by flow cytometry and ELISA. To assess proximal TCR signaling, Jurkat cells were treated with the inhibitor for 1 hour at 37 °C and stimulated with anti-CD3 (5 µg/ml) for 2 minutes. Activation of proximal TCR signaling was assessed by measuring phosphorylation of Lck, ZAP-70 and LAT by immunoblots.

### Luciferase Assay

NFAT-dependent firefly luciferase plasmid was a gift from Dr. Jerry Crabtree (Addgene #17870), AP1-dependent firefly luciferase plasmid was a gift from Dr. Alexander Dent (Addgene #40342), NFκB-dependent firefly luciferase plasmid was a gift from Dr. David Baltimore (Addgene #14886) and a plasmid encoding renilla luciferase was a gift from Dr. Jakob Reiser (CBER/FDA). Jurkat T cells were co-transfected with firefly and renilla luciferase plasmids using the Amaxa Cell Line Nucleofector Kit V, program X-005. Following 24 hours of transfection, cells were either treated with FTY720 (5 µM) or DMSO for 24 hours. Luciferase activity was measured at 24 hours post treatment using Promega’s Dual-Glo Luciferase kit following the manufacture’s protocol. Firefly luciferase activity was normalized to renilla luciferase activity.

### Flow cytometry

Cellular receptor expression was measured using following antibodies S1PR1 (CD363, eFluor 660) from Thermo Fisher, CD3 (V450), CD4 (A700), CD8 (FITC), and CD25 (PE) from BD Biosciences using the manufacturer’s recommendations. Data was acquired on a BD LSR II flow cytometer using single stained CompBeads (BD Biosciences) for compensation. Over 10,000 total events were collected in each experiment and the FlowJo program (Tree Star Inc.) was used for data analysis. All flow cytometry experiments are an average of at least three experiments.

### ELISA

IL-2 and IFN-γ released into cell culture supernatant was quantified using cytokine specific human ELISA kits (BD Biosciences). Arachidonic acid released into the culture supernatant was measured using Human Arachidonic Acid ELISA Kit (Novus Biologicals). ELISAs were performed following the manufacturer’s recommendations.

### Immunoblot Analysis

Cellular lysates were mixed with Laemmli sample buffer, heated at 95 °C for 10 minutes and separated on NuPAGE Bis-Tris gels by electrophoresis and transferred to nitrocellulose membranes using the iBlot transfer system (Thermo Scientific). Membranes were incubated in 3% fat-free dry milk for 1 hour at room temperature followed by overnight incubation with primary antibodies. Proteins were detected with Super Signal West Dura (Thermo Scientific #34075) using a Bio-Rad ChemiDoc MP imaging system. Immunoblots were quantified using ImageJ (NIH). Primary antibodies used were: pLck (Y394, R&D Systems), pZAP-70 (Y319), AP1, NFĸB and Lck from Cell Signaling, pLAT(Y226), total ZAP-70, and NFAT-1 from BD Biosciences, total LAT from Biolegend, beta-Actin from Sigma, CD3 (OKT3), and GAPDH from Thermo Fisher, CD28 from BD Biosciences and acetyl histone H3K9 and trimethyl histone H3K27 from Millipore.

### Statistics

One-way ANOVA was used to compare results from multiple groups and two-sided Student’s t test was used to compare results from two groups. *P* values less than 0.05 were considered statistically significant. GraphPad PRISM software V7.0 (GraphPad Software Inc.) was used for statistical analysis.

### Ethics Statement

All experimental protocols pertaining to PBMCs isolated from human blood are done in accordance with approved guidelines. All experimental protocols were approved by the FDA’s IRB, Research Involving Human Subject Committee (RIHSC).

### Data availability

All the data have been included in the manuscript and the supplementary information.

## Electronic supplementary material

Supplementary Information
