# Supplementary material for: Characterization of the effects of immunomodulatory drug fingolimod (FTY720) on human T cell receptor signaling pathways
Source: Sci Rep. 2018 Jul 19;8:10910. doi: 10.1038/s41598-018-29355-0 (PMC6053412; doi:10.1038/s41598-018-29355-0)

**Characterization of the effects of immunomodulatory drug fingolimod (FTY720) on human T cell receptor signaling pathways**

Alan Baer^1^, Winston Colon-Moran^1^, Nirjal Bhattarai^1^

^1^Division of Cellular and Gene Therapies, Center for Biologics Evaluation and Research, Food and Drug Administration, Silver Spring, Maryland, 20993

**Supplemental Material**

**Supplemental Fig. S1. Phosphorylated FTY720 (pFTY720) does not inhibit T cell receptor (TCR) mediated T cell activation in primary human T cells.** Primary human T cells were treated with DMSO or pFTY720, and following TCR engagement with anti-CD3/CD28, T cell activation was measured by assessing **(b)** IL-2, (**c**) IFN-γ release and CD25 surface expression on (**d**) CD4 and (**e**) CD8 T cells. MFI = mean fluorescence intensity. Data represent the average of three technical replicates, and the standard deviation is shown. Each experiment was independently performed with three different donors with similar results.
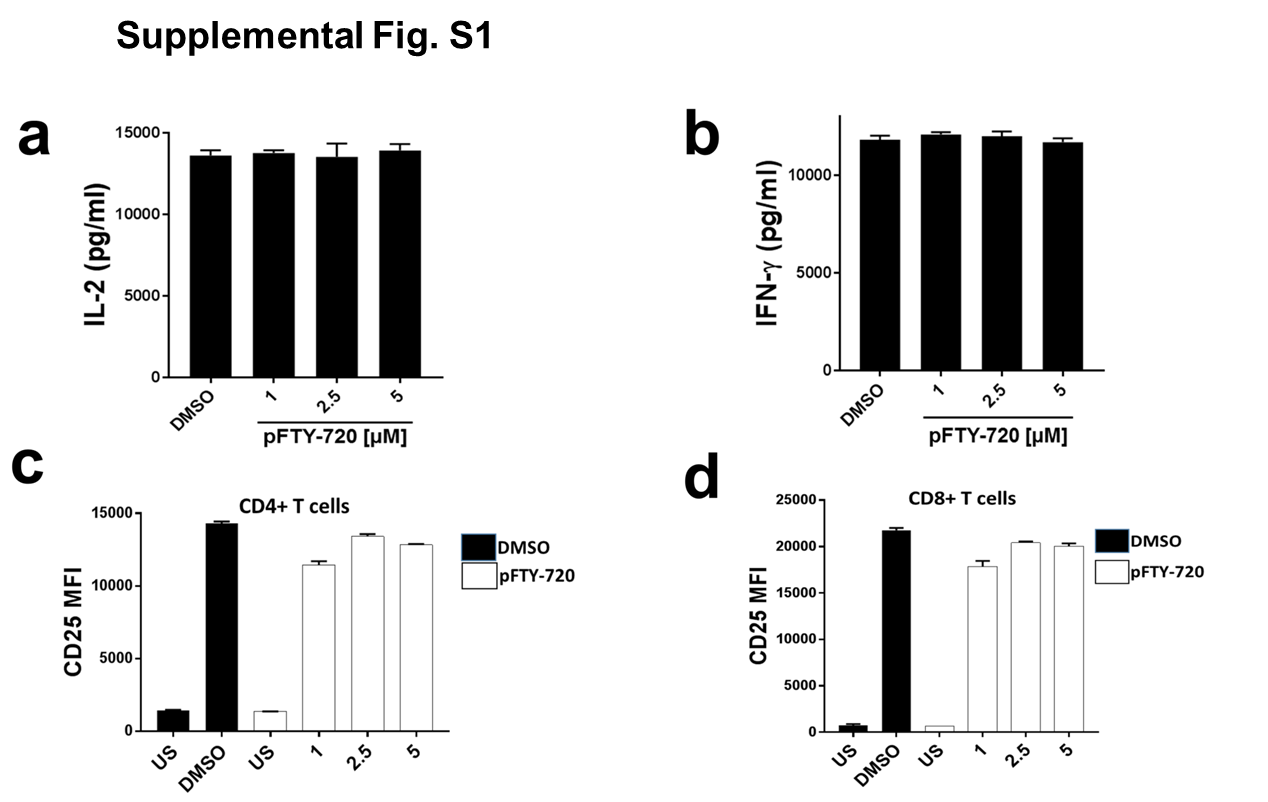


**Supplemental Fig. S2. FTY720 and phosphorylated FTY720 (pFTY720) does not affect T cell activation in resting primary human T cells.** Primary human T cells were treated with DMSO, FTY720 (5µM) or pFTY720 (5µM). Following 18 hours treatment, T cell activation was measured by assessing **(b)** IL-2, (**c**) IFN-γ release and CD25 surface expression on (**d**) CD4 and (**e**) CD8 T cells. MFI = mean fluorescence intensity. Data represent the average of three technical triplicates, and the standard deviation is shown. Each experiment was independently performed with three different donors with similar results.


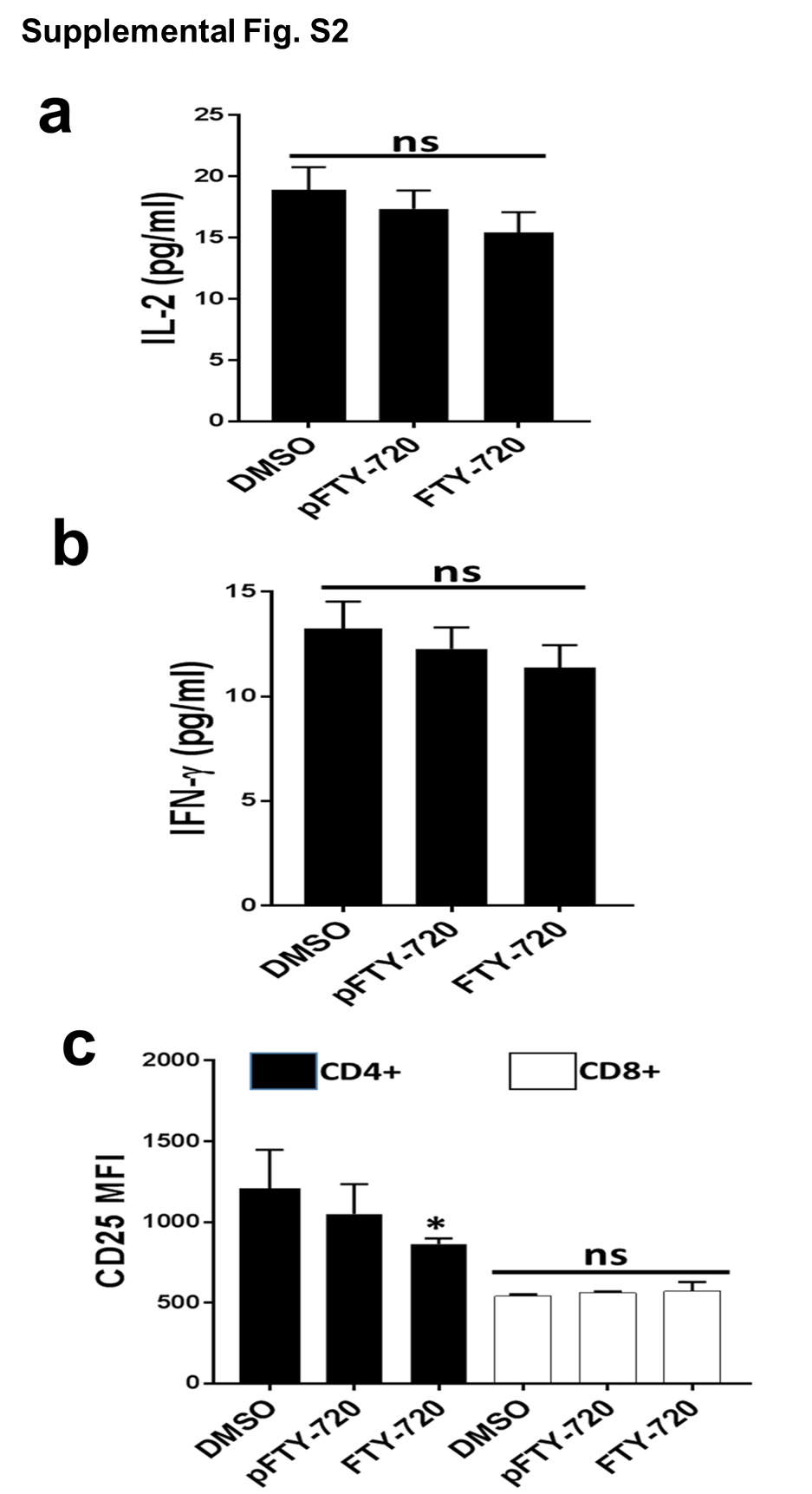


**Supplemental Fig. S3. Calcineurin inhibitors do not affect FTY720 mediated inhibition of T cell activation in primary human T cells.** Primary human T cells were treated with DMSO, FTY720 (5µM) or pFTY720 (5µM) in the presence or absence of two different calcineurin inhibitors, Cyclosporin A (CsA, 5µM) or FK506 (5µM). Following overnight treatment, cells were stimulated with PMA and Ionomycin. T cell activation was measured at 18 hours post stimulation by assessing **(a)** IL-2, and (**b**) IFN-γ release. Data represent the average of three technical triplicates, and the standard deviation is shown. Each experiment was independently performed with three different donors with similar results.


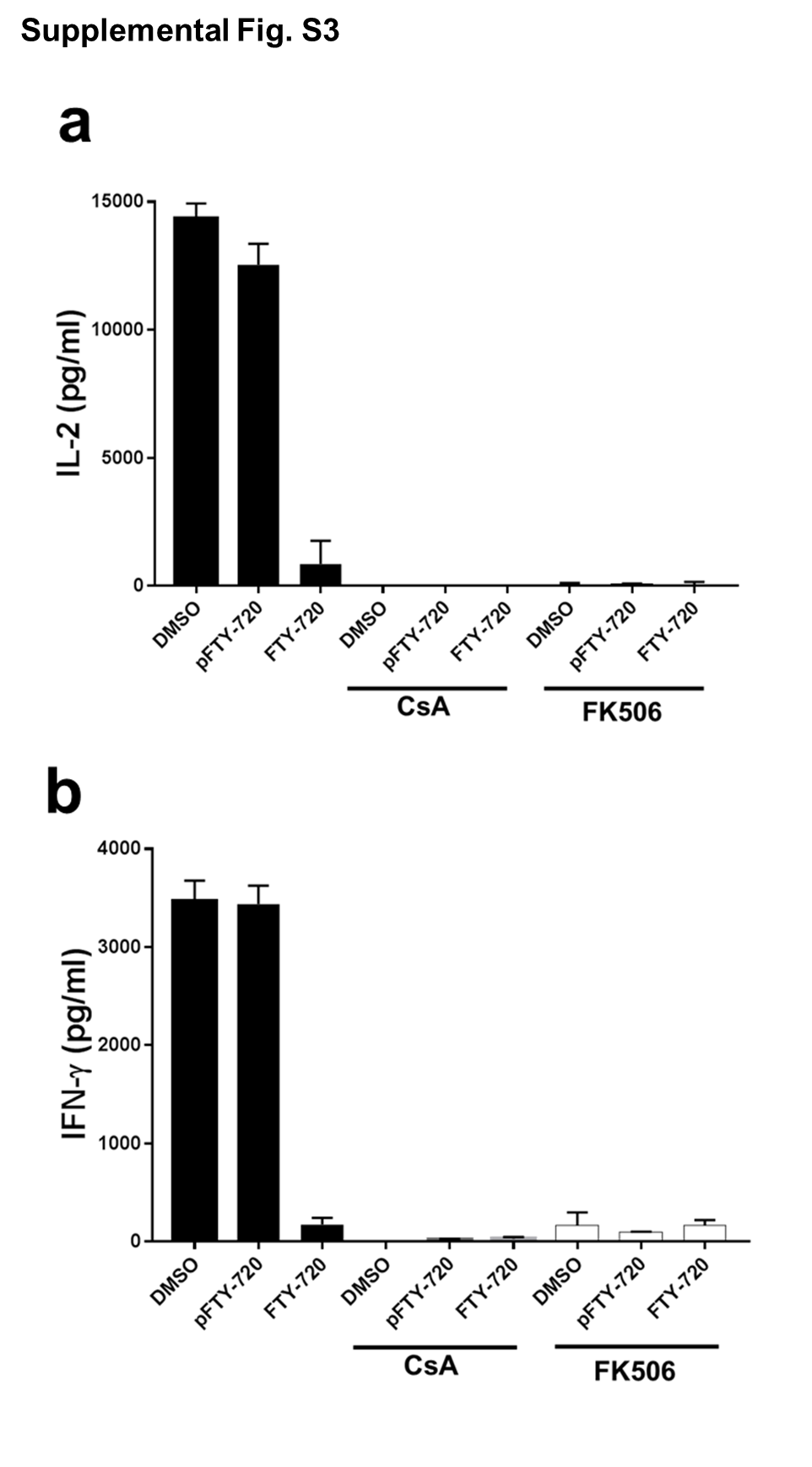


**Supplemental Fig. S4. Phosphorylated FTY720 (pFTY720) and FTY720 inhibit sphingosine-1-phosphate (S1PR1) receptor expression in presence of sphingosine kinase (SphK) inhibitor.** Primary human T cells were treated with DMSO, FTY720 (5µM) or pFTY720 (5µM) in the presence or absence of SphK (5µM). Surface expression of S1PR1 on **(a)** CD4+ and **(b)** CD8+ T cells was measured at 24 hour by flow cytometry. Data represent the average of three technical triplicates, and the standard deviation is shown. Each experiment was independently performed with three different donors with similar results.


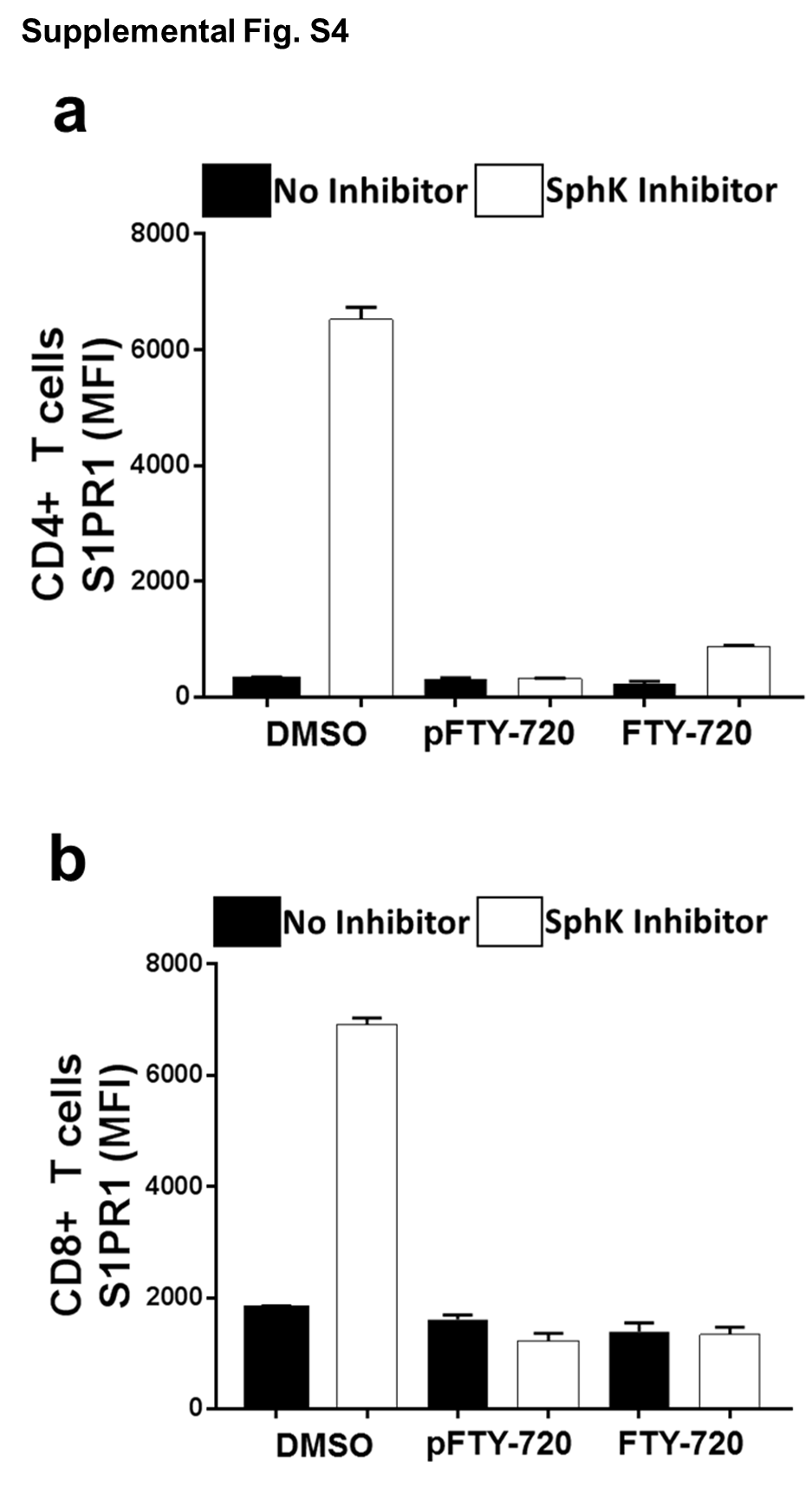


**Supplemental Figure S5:** Immunoblot analysis of nuclear proteins of primary human T cells obtained from two donors treated with DMSO or FTY720 (5 µM) for (a) NFAT-1, (b) NFkB and (c) AP-1 with or without PMA/Ionomycin (P/I) stimulation.

**
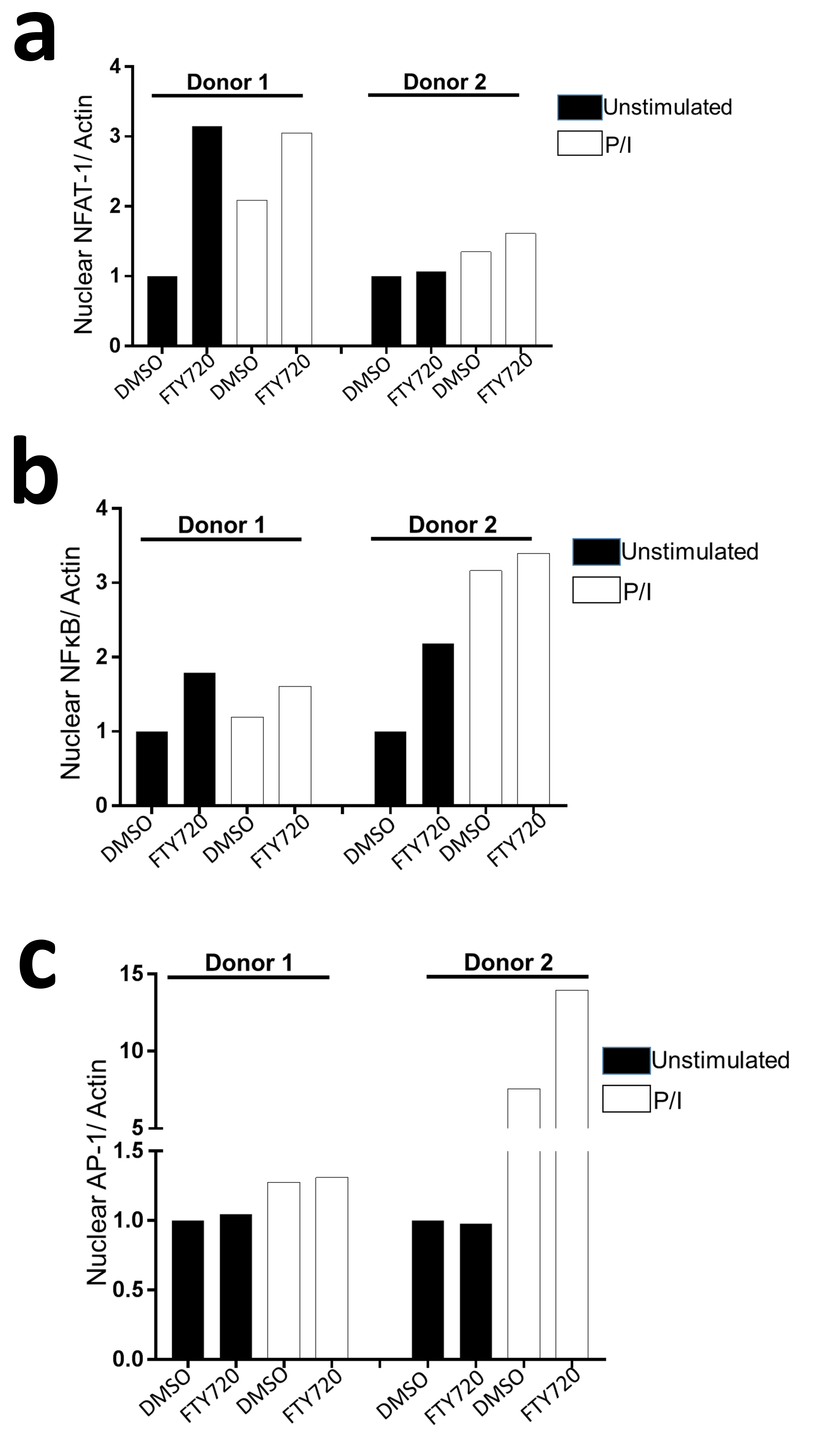
**

**Supplemental Figure S6:** Original membrane image of immunoblot shown in figure 2.


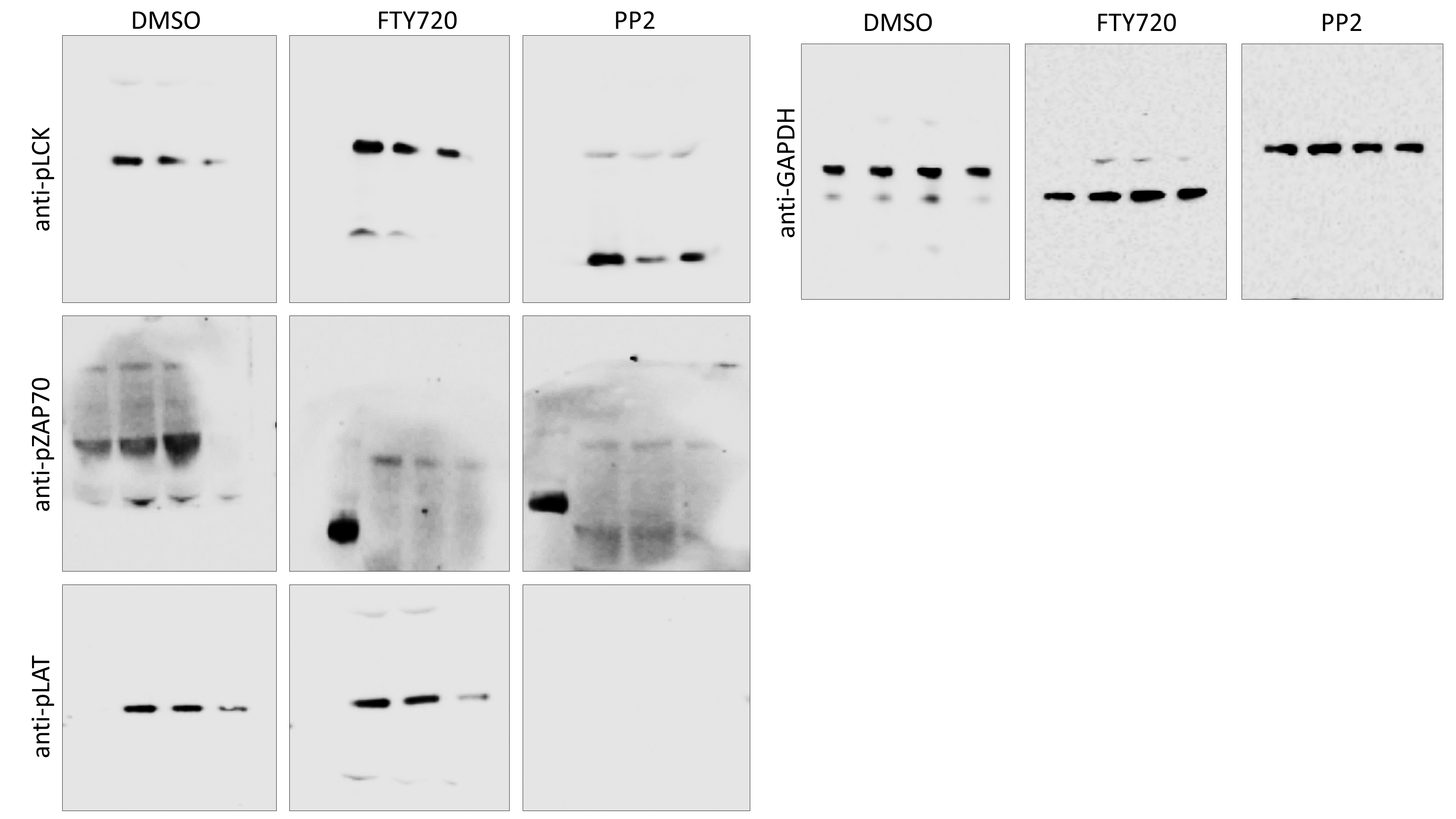


**Supplemental Figure S7:** Original membrane image of immunoblot shown in figure 5e.


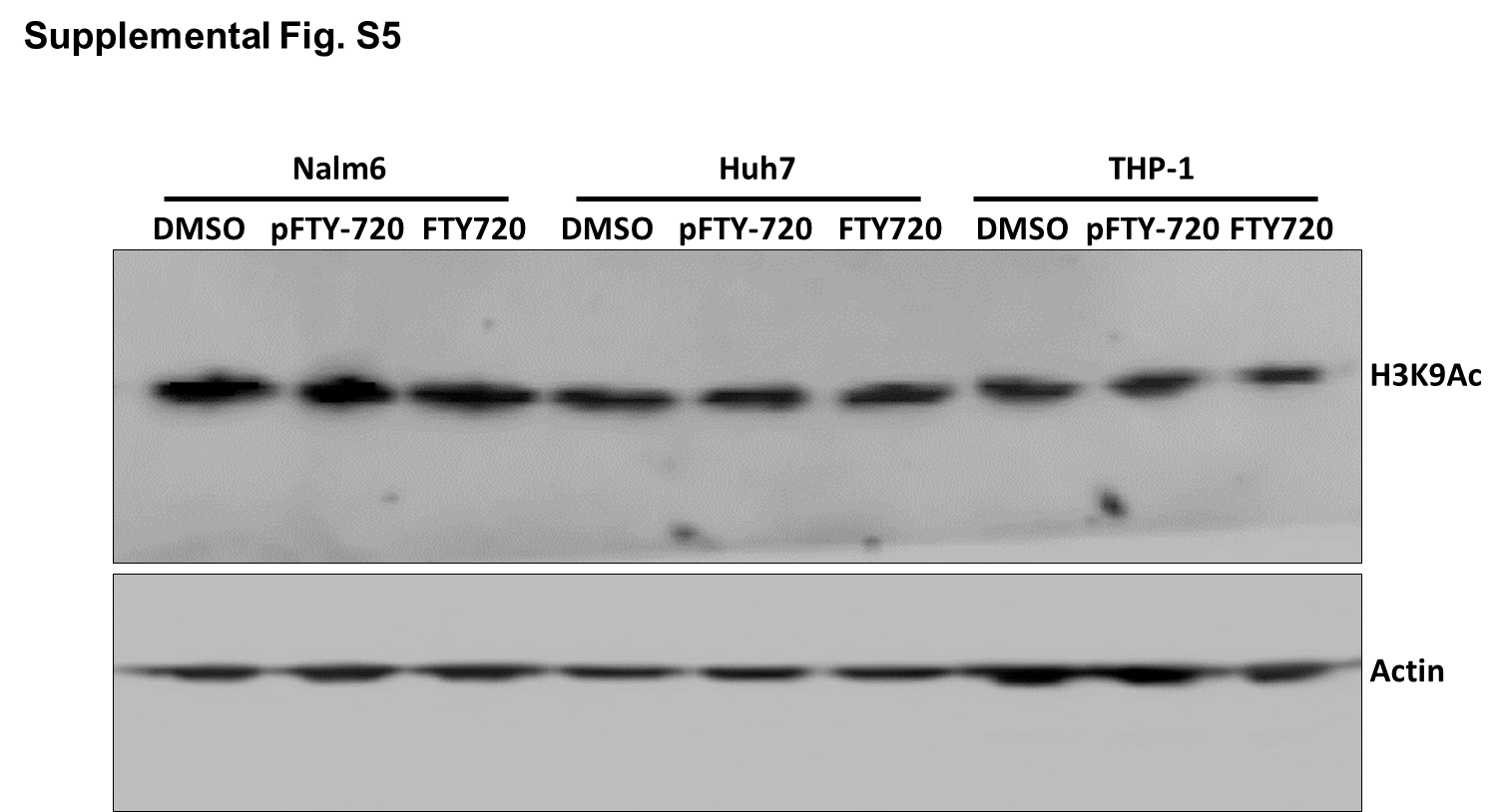

Supplement: Supplementary file 1 — Supplementary Information [file 41598_2018_29355_MOESM1_ESM.doc]
